# Novel and known variants in *GJA3* and *LIM2* in congenital cataract families from North India

**DOI:** 10.1186/s12864-023-09880-7

**Published:** 2024-01-04

**Authors:** Shiwali Goyal, Ravijit Singh, Jai Rup Singh, Vanita Vanita

**Affiliations:** 1 Department of Human Genetics, Guru Nanak Dev University (GNDU), Amritsar, 143005 Punjab India; 2 Dr. Daljit, Singh Eye Hospital, Amritsar, 143001 Punjab India

**Keywords:** ADCC, ARCC, *GJA3*, *LIM2*, Bi-directional sequencing

## Abstract

**Background:**

To identify the underlying genetic defects in autosomal dominant (ADCC) and autosomal recessive (ARCC) congenital cataract families from North India.

**Methods:**

Detailed family histories were collected, pedigrees drawn followed by slit-lamp examination and lens photography. Mutation screening was performed using Sanger sequencing in the known candidate genes for crystallins, connexins, and membrane proteins. The pathogenicity of identified variants was assessed bioinformatically.

**Results:**

In two ADCC families (CC-281 and CC-3015) with posterior lenticonus cataract, a novel change c.263C > T (p.P88L) in *GJA3* in CC-281 family and a previously reported substitution c.388C > T (p.R130C) in *LIM2* in CC-3015 family was observed. In an ARCC family (CC-3005) having central pulverulent cataract, a novel frameshift deletion (c.764delT;p.L255R46fs) in *GJA3* was detected. The observed variants segregated completely with phenotypes in the affected members and were neither present in unaffected family members nor in the ethnically matched 150 controls (tested for two novel variants), hence excluding these as polymorphisms.

**Conclusions:**

Present study identified two novel mutations i.e., c.263C > T;p.P88L and c.764delT;p.L255R46fs in *GJA3* in an ADCC and an ARCC family having posterior lenticonus and central pulverulent cataract, respectively. In another ADCC family with posterior lenticonus cataract, a previously reported mutation c.388C > T;p.R130C in *LIM2* was observed. R130 may be a mutation hotspot as previously ADCC families from different ethnicities (UK/Czechia, China, Spain, Japan) also harbored the same substitution, however, with different phenotypes i.e., nuclear pulverulent, membranous, nuclear, lamellar, and sutural/lamellar. Findings in present study thus expand the mutation spectrum and phenotypic heterogeneity linked with *GJA3* and *LIM2*.

**Supplementary Information:**

The online version contains supplementary material available at 10.1186/s12864-023-09880-7.

## Introduction

Congenital cataract (CC) is characterized as an opacity of the eye lens present at birth or during early childhood that has the potential for inhibiting visual development and may result in permanent blindness [1]. The estimated prevalence of CC is 1–6 cases/10,000 in developed countries and 5–15/10,000 live births in developing nations [2]. According to the World Health Organization, over 14 million children are blind worldwide, with bilateral cataract accounting for more than half of all causes of blindness globally [3]. CC occurs either as an isolated eye anomaly, in association with ocular abnormalities, or as a component of multi-systemic disorders [4]. Approximately 33% of patients have a positive family history and autosomal dominant is the commonest mode of inheritance, however, autosomal recessive, and X-linked modes also exist [5, 6]. CC exhibits extensive genetic heterogeneity with > 50 loci and mutations in > 35 genes at these loci identified for non-syndromic types (https://cat-map.wustl.edu). Mapped genes at these loci mainly include genes for crystallins, lens-specific connexins, cytoskeletal structural proteins, major intrinsic proteins or aquaporins, paired-like homeodomain transcription factor 3, avian musculoaponeurotic fibrosarcoma, heat shock transcription factor 4, and transmembrane proteins (https://cat-map.wustl.edu).

The major structural and functional lens-specific proteins are crystallins which make up to 90% of water-soluble proteins of the mammalian lens. Crystallins are well-organized and provide a refractive index gradient, which allows for lens transparency [7]. Mutations in crystallins or other lens proteins result in protein aggregation, which leads to cataract [8]. Gap junctions (GJs)/connexins (Cx) are crucial for the development of the complex intercellular communication system that maintains the metabolic balance and [9] helps in the movement of ions and small molecules ~ 1 kDa between the cells [10]. Connexins belong to a multigene family with at least 21 members and have intricate and overlapping expression patterns [11]. Cx43 (GJA1), Cx46 (GJA3), and Cx50 (GJA8) are essential for binding fiber and epithelial cells together in the lens as a syncytium [12]. The ocular defects and cataractogenesis linked with mutations in Cx46 and Cx50 serve as evidence for the significance of GJs for lens physiology. The mechanisms implicated in the development of different phenotypes of CC mainly include altered voltage-dependent gating and permeability features, gain of hemichannel function, reduced trafficking to the plasma membrane, and dominantly negative effects of mutant alleles over wild-type connexins [13].

The second-most abundant integral membrane protein in the ocular lens fiber cells of vertebrates is LIM2/MP19 (Mol. wt. 19 kDa). It is mostly localized in the junctional portions of the lens fiber cell membrane as well as other fiber cell membranes, indicating a function in the lens junctional communication [14]. Mature as well as developing lens fiber cells are reported to express MP19 more prominently than proliferating epithelial cells in the lens [15]. It binds to the lens cell membrane protein galectin-3 [16] as well as the calmodulin [17] to form homo-oligomers and may play a role in gap junction formation.

The present study was performed to identify the underlying genetic defects in two autosomal dominant congenital cataract (ADCC) families (CC-281 and CC-3015) both with bilateral posterior lenticonus cataract, and an autosomal recessive congenital cataract (ARCC) family (CC-3005) having bilateral central pulverulent cataract from North India. A novel frameshift deletion c.764delT (p.L255R46fs) in the CC-3005 family in *GJA3* has been identified. In CC-281 a novel variant c.263C > T (p.P88L) in *GJA3* has been observed, whereas in CC-3015 family an already reported mutation c.388C > T (p.R130C) in *LIM2* has been detected. These findings thus expand the mutation spectrum and highlight the phenotypic heterogeneity with *GJA3* and *LIM2*.

## Materials and methods

Written informed consent was obtained from each participant (from legal custodians for minors). Present study got approval from Institutional Ethics Committee (Ref number 1070HG/24.6.2015) of Guru Nanak Dev University (GNDU), Amritsar, India, consistent with the Declaration of Helsinki.

### Family description

An ARCC family (CC-3005) and two ADCC families (CC-3015 and CC-281) were recruited from Northern India at Dr. Daljit Singh Eye Hospital, Amritsar. The clinical and demographic details of individuals recruited in the present study were taken. 5–10 ml of venous blood were collected in EDTA tubes and genomic DNA got isolated [18].

### Mutation screening

In congenital cataracts, about half of the identified mutations are detected in crystallin genes, a quarter in connexin genes, and some in genes for membrane proteins. Therefore, mutation screening was performed in the exonic regions and exon–intron boundaries of the candidate genes for crystallins (*CRYαA, CRYαB*, *CRYβA1/A3, CRYβA2, CRYβA4, CRYβB1, CRYβB2, CRYβB3, CRYγA, CRYγB, CRYγC, CRYγD,* and *CRYγS*); connexins (*GJA3* and *GJA8*) and lens membrane specific genes (*LIM2* and *MIP*) using region-specific primers. Initially, genomic DNA from an affected and an unaffected individual from each of the families was amplified. The amplified products were tested on a 2%-2.5% agarose gel and purified using a QIAquick PCR purification kit (Catalog number 28104). Using BigDye^TM^Terminator Cycle Sequencing Kit ver.3.1 (ABI, Foster City, CA, United States), purified PCR products were sequenced bi-directionally. The sequencing reaction products were purified using 75% isopropanol precipitation method (ABI protocol), resuspended in 10 μl Hi-Di formamide (ABI, Foster City, CA, USA), denatured at 95 °C for 5 min, and electrophoresed on ABI 3500xL Genetic Analyzer (Applied Biosystems, ThermoFisher Scientific). Sequences were assembled and analyzed using SeqMan II program of the Lasergene package (DNASTAR Inc., Madison, WI). Upon identification of nucleotide substitutions in *GJA3* (proband IV:3; CC-3005 family and proband IV:5; CC-281 family) and in the *LIM2* (proband III:2; CC-3015 family), other available affected and unaffected family members were tested for the observed sequence variants to see their co-segregation with the phenotypes. Further, 150 ethnically matched controls (free from any eye anomaly) were tested for the observed novel variants in *GJA3* (c.263C > T and c.764delT) to exclude their possibility as polymorphisms.

### In silico analysis

The identified variants c.263C > T (p.P88L;CC-281) and c.764delT (p.L255R46fs;CC-3005) in *GJA3* and c.388C > T (p.R130C;CC-3015) in *LIM2* were interpreted using HOPE software (https://www.cmbi.umcn.nl/hope), and VarCards (http://var-cards.biols.ac.cn/). Varcards is in agreement with guidelines of American College of Medical Genetics. Additionally, cross-species conservation analyses were performed using HomoloGene NCBI (https://www.ncbi.nlm.nih.gov/homologene). Transmembrane protein display was done using TOPO2 software (http://www.sacs.ucsf.edu/TOPO2/).

## Results

### Phenotype description

**Fig. 1:**
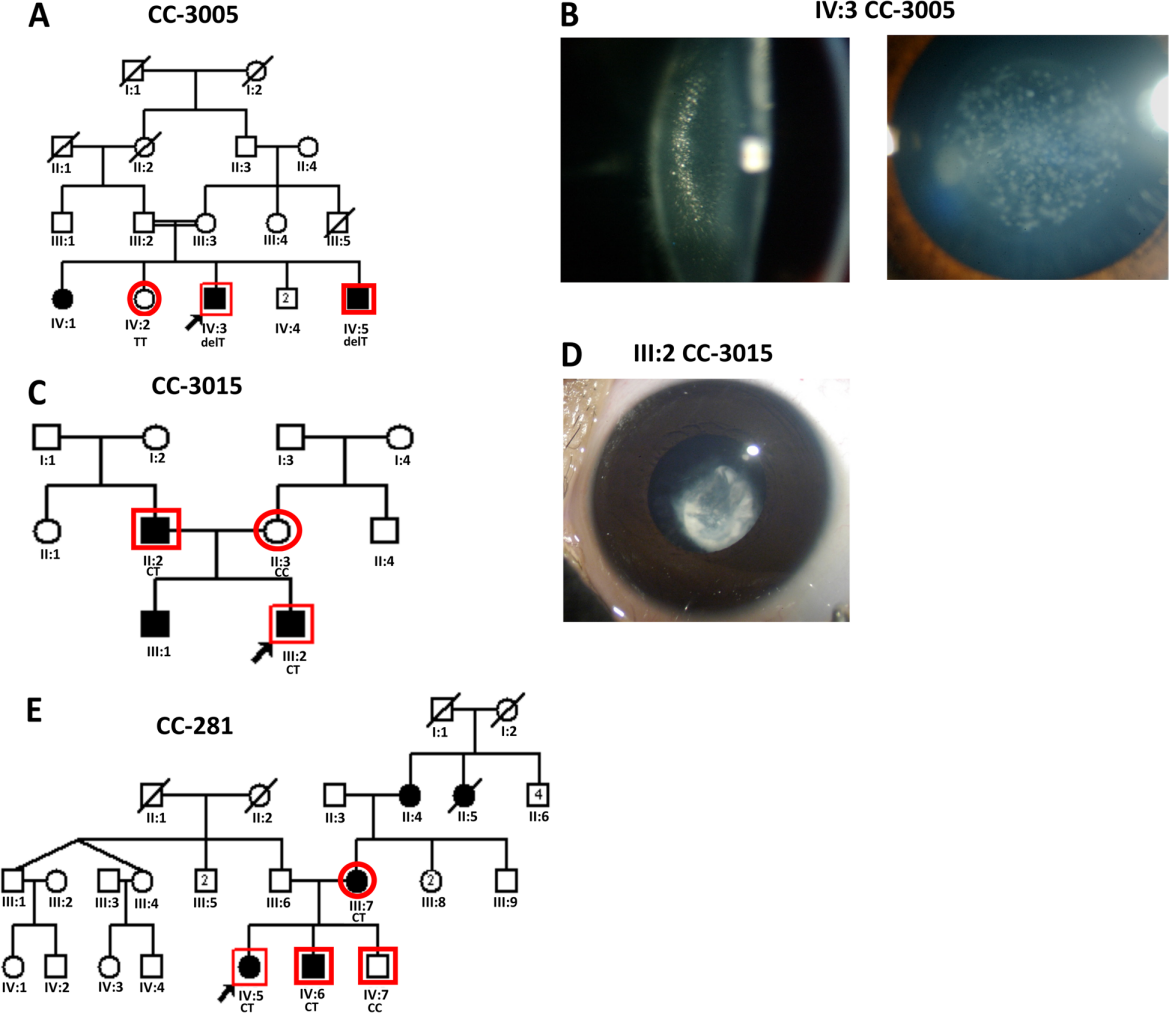
**A** Pedigree of a four-generation autosomal recessive congenital cataract family (CC-3005) indicating three affected individuals (IV:1, IV:3, and IV:5) in a single sibship and their parents (III:2 and III:3) being first cousins. The proband (IV:3) is indicated with an arrow. **B** Slit-lamp and lens photographs of the left eye of the proband (IV:3) of the CC-3005 family indicating central pulverulent cataract. **C** Pedigree of an autosomal dominant congenital cataract family (CC-3015) indicating three affected individuals in two generations. The proband (III:2) is indicated with an arrow. **D** Photograph of the eye lens of the proband (III:2) of the CC-3015 family indicating posterior lenticonus cataract. **E** Pedigree of an autosomal dominant congenital cataract family (CC-281) indicating five affected individuals in three generations. The proband (IV:5) is indicated with an arrow. Squares and circles symbolize males and females, respectively. Symbols marked in red indicate the individuals who participated in the present study by giving blood samples and undergoing ophthalmic examination

The proband (IV:3) in autosomal recessive family (CC-3005) (Fig. 1A) had bilateral central pulverulent cataract (Fig. 1B). Slit-lamp examination revealed that the cataract involved the larger fetal nucleus. The opacities in the central embryonic zone were rarer and these increased in density towards the periphery resembling the zonular pulverulent cataract. He underwent cataract removal (both eyes [B/E]) at the age of ~ 11 years. His best corrected visual acuity (BCVA) was 6/18 in B/E, at his last visit. His younger brother (IV:5) was also diagnosed with a central pulverulent cataract at the age of ~ 3 years and underwent cataract surgery at ~ 11 years of his age. His post-operative BCVA was 6/18 in B/E. Their elder sister (IV:1) who also had a history of bilateral cataract since childhood, however, was not available for the present study.

The proband (III:2) in an autosomal dominant family (CC-3015) (Fig. 1C) was diagnosed with posterior lenticonus cataract at the age of ~ 4 years. On ocular examination, an almond-shaped lenticular opacification in the central 4-5 mm zone, involving posterior subcapsular region (Fig. 1D) was observed. It showed areas of clearer zones interspersed with denser opaque regions. The posterior surface of the lens showed a conical projection towards the vitreous cavity. His father (II:2) and elder brother (III:1) affected with bilateral CC were already operated for B/E in the first decade of their lives. The proband (III:2) got operated for cataract (B/E) at the age of ~ 9 years. In the proband (III:2), cataract extraction and intraocular lens implantation recovered the vision to 6/18 in B/E.

The proband (IV:5) a 7–10 year old female (Fig. 1E) in another autosomal dominant family (CC-281) was diagnosed and operated for bilateral posterior lenticonus cataract (lens photo unavailable). Her BCVA was 6/12 and 6/18 in the right and left eye, respectively, after the cataract surgery. Her mother (III:7), grandmother (II:4), and grandmother’s sister (II:5), had got operated for CC (B/E) in their first decades. Her younger brother (IV:6) got operated at the age of ~ 3 years, and his BCVA after surgery was 6/12 in B/E.

### Mutation screening

**Fig. 2:**
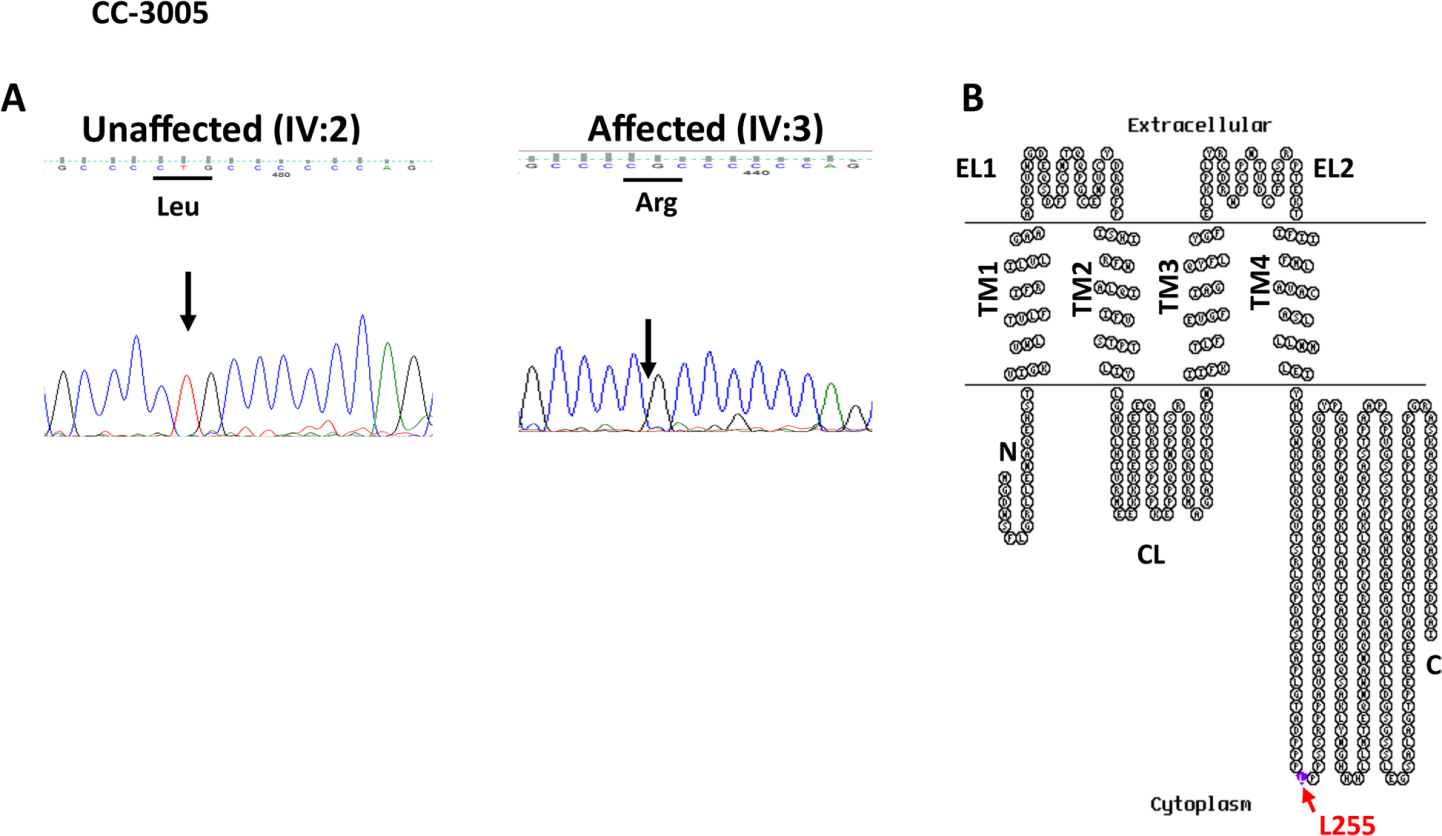
**A** Electropherograms of a part of the forward strand sequence of exon 1 of *GJA3* in an unaffected (IV:2) and an affected individual (IV:3) of the CC-3005 family. In the unaffected individual (IV:2) arrow indicates the wild-base T at c.764 bp position whereas in the affected individuals (IV:3 and IV:5) arrow indicates the nucleotide position at which deletion of base T occurred that resulted in c.764delT (p.L255R46fs). **B** The membrane topological structure of GJA3 was generated by TOPO2 software. The L255 (indicated by a purple dot) is localized in the cytoplasmic domain

In ARCC (CC-3005) family bidirectional sequence analysis of the selected candidate genes (*CRYαA, CRYαB*, *CRYβA1/A3, CRYβA2, CRYβA4, CRYβB1, CRYβB2, CRYβB3, CRYγA, CRYγB, CRYγC, CRYγD, CRYγS, GJA3 GJA8, LIM2*, and *MIP*) showed a novel change in *GJA3* i.e., c.764delT (Fig. 2A) in homozygous form in the proband (IV:3), as well as in his affected sibling (IV:5). This deletion was not seen in their unaffected sister (IV:2) nor in 150 unrelated controls from the same ethnicity, hence excluding its possibility as a polymorphism. The deletion c.764delT replaced leucine with arginine at codon 255 and caused a frameshift (p.L255R46fs) resulting in termination codon forty-six amino acids downstream. Leu255 resides in the cytoplasmic loop of connexin 46 (Fig. 2B).

**Fig. 3:**
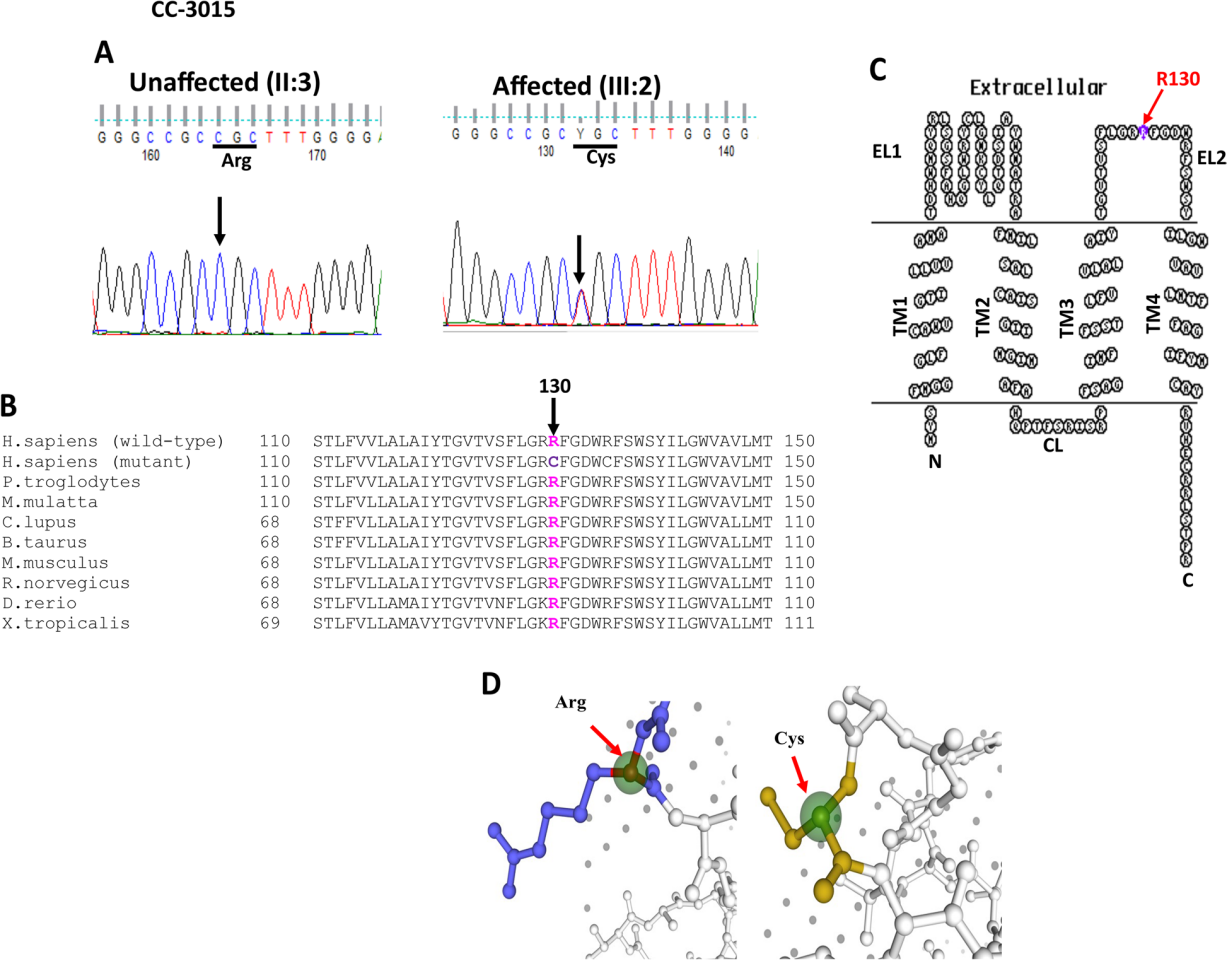
**A** Electropherograms of a part of the forward strand sequence of exon 4 of *LIM2* in an unaffected individual (II:3) and in the affected individual (III:2) of the CC-3015 family. In the unaffected individual (II:3) arrow indicates the position of wild-type base C, whereas in the affected individuals (II:2 and III:2) arrow indicates the nucleotide at which heterozygous change (CT) occurred that resulted in c.388C > T (p.R130C). **B** Multiple amino acid sequence alignment (NCBI HomoloGene) of LIM2 indicating conservation of arginine [R] (shown in pink) at position 130 as indicated by an arrow in different species. The mutant sequence (Homo sapiens [mutant]) (replacement of arginine [R] by cysteine [C] at 130 position) in the affected members is highlighted in purple color. **C** The membrane topological structure of LIM2 generated by TOPO2 software indicated that the mutation p.R130C (indicated by a purple dot) is located in the extracellular loop 2 (EL2). **D** 3-D structure of LIM2 drawn using swiss-pdb software, indicated wild-type arginine to be bigger in size as compared to the mutant cysteine

In an ADCC (CC-3015) family bidirectional sequence analysis of the selected candidate genes indicated c.388C > T change in *LIM2* segregating in the heterozygous form (CT) in the proband (III:2) (Fig. 3A). This alteration was observed in affected father (II:2) of the proband too, and not seen in his unaffected mother (II:3). The c.388C > T substitution replaced arginine with cysteine at amino acid position 130 (p.R130C) which is evolutionarily highly conserved in different species (Fig. 3B). The R130C change lies in the second extracellular loop of the LIM2 (Fig. 3C). HOPE predicted wild-type arginine bigger in size as compared to the mutant cysteine (Fig. 3D). Due to p.R130C substitution, the charge of the wild-type residue will be lost, which can cause loss of interactions with other residues or molecules.

**Fig. 4:**
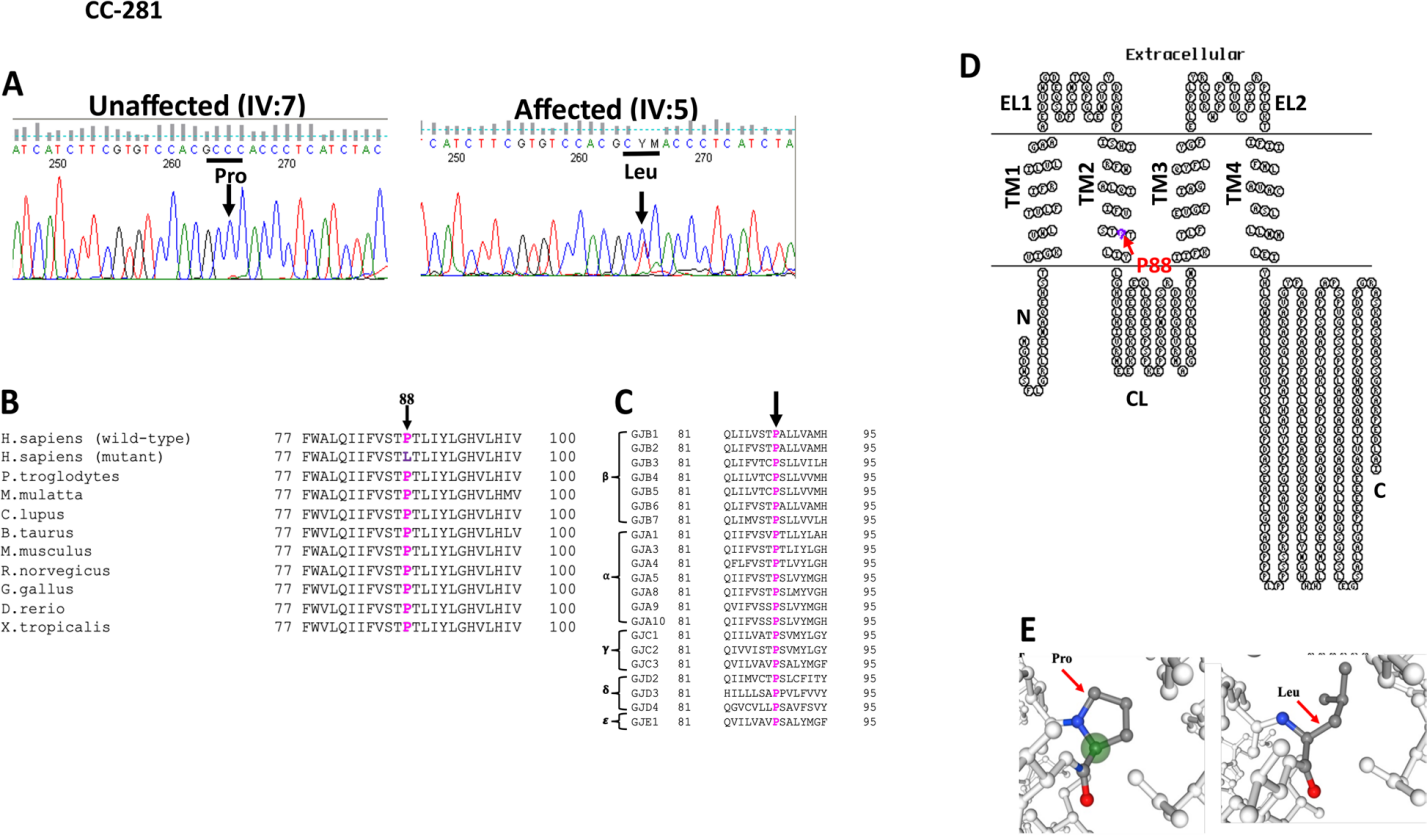
**A** Electropherograms of a part of the reverse strand sequence of exon 1 of *GJA3* in the unaffected individual (IV:7) and affected individual (IV:5) of the CC-281 family. In the unaffected individual (IV:7) arrow indicates wild-type base C, whereas in the affected individuals (III:7, IV:5, and IV:6) arrow indicates the nucleotide at which heterozygous change (CT) occurred that resulted in c.263C > T (p.P88L). **B** Multiple amino acid sequence alignment (NCBI HomoloGene) of GJA3 indicating conservation of proline [P] (shown in pink) at position 88 as indicated by an arrow in different species. Mutant sequence (Homo sapiens [mutant]) (replacement of proline [P] by leucine [L] at 88 position) in the affected members of the present analyzed CC-281 family is highlighted in purple color. **C** Proline at position 88 (indicated by pink) is highly conserved in different human gap junction (connexins) proteins as well. **D** The membrane topological structure of GJA3 generated by TOPO2 software indicated that mutation p.P88L (indicated by a purple dot) is localized in the second transmembrane domain. **E** 3-D structure of GJA3 drawn using swiss-pdb software, indicated mutant leucine is bigger in size as compared to the wild-type proline

In another ADCC (CC-281) family bidirectional sequence analysis of the selected candidate genes indicated c.263C > T change in *GJA3* segregating in heterozygous form (CT) in the proband (IV:5) (Fig. 4A)*,* and in other two available affected members (IV:6 and III:7). This alteration was neither seen in the unaffected sibling (IV:7) nor in the 150 ethically matched unrelated controls, thus excluding it as polymorphism. The c.263C > T substitution replaced proline with leucine at amino acid position 88 (p.P88L) which is evolutionarily highly conserved in different species (Fig. 4B) and in different human connexins (Fig. 4C). p.P88 is located in the second transmembrane domain (Fig. 4D) of connexin 46. HOPE predicted that the mutant residue is bigger than the wild-type residue (Fig. 4E), and this size difference may affect the contacts of mutant protein with the lipid membrane.

Different bioinformatics tools predicted both the missense changes i.e., c.263C > T (p.P88L) (Supplementary Table 1) in *GJA3* in family CC-281 and c.388C > T (p.R130C) in *LIM2* (Supplementary Table 2) in family CC-3015 to be damaging/disease-causing/deleterious/pathogenic. The minor allele frequency (MAF) of c.263C > T (p.P88L; rs1299066109) variant in *GJA3* for South Asians was neither reported in the 1000 genome database (1000G; http://www.1000genomes.org/), gnomAD (https://gnomad.broadinstitute.org/) nor in the dbSNP, indicating this to be a novel variant. Similarly, the MAF of c.764delT variant (rs757100428) in *GJA3* for South Asians in the gnomAD is 0.00004234, however, its MAF has not been reported in the 1000G, nor in the dbSNP, indicating this to be a rare variant. MutationTaster and SIFT predicted c.764delT to be disease-causing and damaging.

## Discussion

In the present study, in an ARCC (CC-3005) family with central pulverulent cataract, we have identified a novel deletion mutation c.764delT (p.L255R46fs) in *GJA3*. Another novel missense variant c.263C > T (p.P88L) in *GJA3* has been observed in an ADCC (CC-281) family with posterior lenticonus cataract. In the second ADCC (CC-3015) family with posterior lenticonus cataract we have observed a known missense mutation c.388C > T (p.R130C) in *LIM2*. These substitutions segregated completely with the disease in the affected members in the respective families and were not seen either in the available unaffected family members nor in the 150 ethnically matched controls (tested for novel variants p.P88L and p.L255R46fs) in *GJA3*, hence excluding these as polymorphisms.

Cx46/GJA3 harbor four transmembrane domains (TM1-TM4), connected by two extracellular loops (EL1-EL2) and a cytoplasmic (CL) loop with NH_2_- and COOH- termini (Fig. 2B and 4D). The extracellular loops are the most conserved domains among connexins and are thought to be involved in the interaction between connexons in adjacent cells. Transmembrane domains (TM1-TM4) perform a variety of functions i.e., enzyme catalysis, transport across membranes, transducing signals as receptors of hormones and growth factors, and energy transfer in ATP synthesis. Cytoplasmic loop controls the activity and permeability properties of the channels [19].

Cx46 is essential for coupling fiber cells, particularly mature fiber cells in the central core of the lens. Deletion of the *GJA3* gene resulted in severe nuclear cataract in mice [20]. To date 70 mutations have been reported in *GJA3*, of which 67 mutations are associated with isolated cataracts and only three are associated with syndromal cataracts (https://cat-map.wustl.edu/). Diverse phenotypes have been reported in cataract patients harboring mutations in *GJA3*. Most common phenotype is nuclear, followed by cortical, and the remaining are capsular, lamellar, sutural, or subcapsular. Interestingly, most mutations in EL1 and EL2 domains of Cx46 result in nuclear-type cataracts, whereas mutations in the CL domain result in either nuclear or cortical phenotypes. In the present analyzed ARCC family (CC-3005) affected members harboring deletion mutation c.764delT (p.L255R46fs) at codon 255 localized in the cytoplasmic loop (Fig. 2B) had central pulverulent cataract (also known as nuclear cataract). This further supports the fact that mutations in the CL domains are linked with nuclear cataract. The MAF of c.764delT (p.L255R46fs; *GJA3*) in South Asians, in gnomAD is 0.00004234, and no MAF is documented in the 1000G or in the dbSNP, thus indicating this as a rare variant. Interestingly, 100 unaffected controls from a Pakistani population also carried wild-type amino acid leucine (p.L255) at this position (personal communication, J. Brink, June 1, 2023). Additionally, SIFT and MutationTaster predicted this change to be damaging and disease-causing.

In another ADCC family (CC-281) with posterior lenticonus cataract, another novel change i.e., c.263C > T in *GJA3* has been observed. The c.263C > T substitution resulted in the replacement of highly conserved proline with leucine at codon 88 (p.P88L) (Fig. 4B and C) that is localized in the second transmembrane domain (TM2) of the connexin 46 (Fig. 4D). This indicates that proline at position 88 in Cx46 is of functional significance, and its replacement with another residue may have a detrimental physiological effect. Cx46 and Cx50 form heteromeric/heterotypic intercellular channels in the mammalian lens [21, 22]. Interestingly, in connexin 50/GJA8 in the TM2 the substitutions of proline at identical codon i.e., P88 with different amino acids (P88S, P88Q, P88T, P88L) linked with zonular pulverulent/nuclear, lamellar, total, and Balloon-like (in an Indian family identified by us) cataract, have previously been reported [23–27]. Pal et al. [28] documented that the P88S mutant in the connexin 50 functions in a dominant-negative way and completely abolishes the function of the gap junction channel. Interestingly, Jin et al. [26] identified p.P88L mutation in the Cx50 (as similarly, observed in the present Indian family, however in Cx46) in a four-generation Chinese pedigree with congenital dominant cataract. Authors further reported that P88 is located at the homo/heteromeric interface but not the homo/heterotypic interface, hence the p.P88L is likely to disrupt the interactions at the homo/heteromeric interface or the Cx50 conformation. The prolines are known to be very rigid and, therefore, induce a spacial backbone conformation which might be required at this position (Pro88), and the observed mutation c.263C > T (p.P88L) in Cx46, in our analyzed ADCC family may disturb this spacial conformation.

In second ADCC family (CC-3015) with posterior lenticonus cataract, a missense variant c.388C > T (p.R130C) has been observed in the *LIM2*. LIM2 is the second most abundant integral membrane protein in vertebrates' ocular lens fiber cells. It has four transmembrane domains, two extracellular loops, a cytoplasmic loop with amino and carboxyl termini [29]. As LIM2 is localized at junction of the lens fiber cells and fiber cell membrane, it indicates its role in the junctional communication [14, 30]. Of all the mutations reported so far in *LIM2*, R130C is the most common one present across different ethnic groups, (https://cat-map.wustl.edu/), therefore, indicating it to be a mutation hotspot. This p.R130C has previously been reported with different phenotypes i.e., nuclear pulverulent, membranous, nuclear, lamellar, and sutural/lamellar in autosomal dominant families from different regions (UK/Czechia, China, Spain, Japan) [31]. However, the phenotype observed in our analyzed Indian family is different i.e., posterior lenticonus cataract, indicating phenotypic variability linked with p.R130C mutation. The R130C substitution lies in the second extracellular loop of LIM2 protein (Fig. 3C) and is likely to disturb membrane trafficking and fiber cell–cell communication. Since disulfide bridges are crucial to the first extracellular loop's functionality, the presence of another cysteine in the extracellular loop 2 (R130C) may prevent the tetraspanin homology domain from folding properly [31]. Different bioinformatic algorithms predicted c.388C > T (p.R130C) in *LIM2* to be disease-causing and highlighted R130 to be evolutionarily conserved. The mutant residue (cysteine130) is predicted to be smaller than the wild-type amino acid arginine (Fig. 3D), which might lead to a loss of interactions. Also, the mutation p.R130C introduced a more hydrophobic cysteine at codon 130, which can result in loss of hydrogen bonds and/or disturb correct folding.

In summary, we described a novel mutation c.263C > T (p.P88L) in *GJA3* in an ADCC family (CC-281) with posterior lenticonus cataract. In second ADCC family (CC-3015) having posterior lenticonus cataract, a previously reported c.388C > T (p.R130C) mutation in *LIM2* has been detected. R130 could be a mutation hot-spot as previously different ADCC families from different ethnicities (UK/Czechia, China, Spain, Japan) with a range of phenotypes have been reported to harbour this substitution. We also observed a novel single base deletion c.764delT (p.L255R46fs) in *GJA3* in an ARCC family (CC-3005) with central pulverulent cataract. The mutations identified in the present study thus expand the mutation spectrum and highlight the inherent phenotypic heterogeneity linked with *GJA3* and *LIM2* for congenital cataract.

### Supplementary Information

**Additional file 1: Supplementary Table 1. **Functional prediction results of c.263C>T (p.P88L) in *GJA3* by various software programs and arithmetics.

**Additional file 2 Supplementary Table 2.** Functional prediction results of c.388C>T (p.R130C) in *LIM2* by various software programs and arithmetics.

## Data Availability

The datasets generated and/or analysed during the current study are available in the DNA Data Bank of Japan (DDBJ) repository with accession numbers LC730524 and LC730525.
