# Supplementary material for: Novel and known variants in GJA3 and LIM2 in congenital cataract families from North India
Source: BMC Genomics. 2024 Jan 4;25:31. doi: 10.1186/s12864-023-09880-7 (PMC10765620; doi:10.1186/s12864-023-09880-7)
Supplement: Supplementary file 2 — Additional file 2 Supplementary Table 2. Functional prediction results of c.388C>T (p.R130C) in LIM2 by various software programs and arithmetics. [file 12864_2023_9880_MOESM2_ESM.docx]

**Supplementary Table 2. Functional prediction results of c.388C>T (p.R130C) in *LIM2* by various software programs and arithmetics**

| **Algorithm** | **Score** | **Prediction** | **Damaging score criteria** |
| --- | --- | --- | --- |
| SIFT | 0.001 | Damaging | ≤0.05 damaging; >0.05 tolerable |
| Polyphen-2_HDIV | 1 | Probably_damaging | ≥0.453 probably damaging; <0.453 tolerable |
| Polyphen-2_HVAR | 1 | Probably_damaging | ≥0.447 probably damaging; <0.447 tolerable |
| LRT | 0 | Deleterious | ≤0.001 deleterious; >0.001 tolerable |
| MutationTaster | 1 | Disease_causing | >0.5 disease causing; ≤0.5 tolerable |
| MutationAssessor | 2.32 | Damaging | >1.9 damaging; ≤1.9 tolerable |
| FATHMM | -2.5 | Damaging | ≤1.5 damaging; >1.5 tolerable |
| PROVEAN | -4.06 | Damaging | ≤2.5 damaging; >-2.5 tolerable |
| VEST3 | 0.921 | Damaging | ≥0.5 damaging; <0.5 tolerable |
| MetaSVM | 0.794 | Damaging | >0 damaging; ≤0 tolerable |
| MetaLR | 0.81 | Damaging | >0.5 damaging; ≤0.5 tolerable |
| M-CAP | 0.118 | Damaging | >0.025 damaging; ≤0.025 tolerable |
| CADD | 35 | Damaging | >20 damaging; ≤20 tolerable |
| DANN | 0.999 | Damaging | ≥0.99 damaging; <0.99 tolerable |
| FATHMM_MKL | 0.854 | Damaging | >0.5 damaging; ≤0.5 tolerable |
| Eigen | 0.729 | Damaging | ≥0 damaging; <0 tolerable |
| GenoCanyon | 1 | Damaging | >0.999 damaging; ≤0.999 tolerable |
| fitCons | 0.554 | Tolerable | >0.7 damaging; ≤0.7 tolerable |
| GERP++ | 4.73 | Conserved | ≥2 DNA sequence is conserved |
| phyloP | 5.974 | Conserved | >2 DNA sequence is conserved |
| pastCons | 1 | Conserved | >0.999 DNA sequence is conserved |
| SiPhy | 15.224 | Conserved | ≥12 DNA sequence is conserved |
| REVEL | 0.885 | Damaging | ≥0.4 is damaging; <0.4 tolerable |
| ReVe | 0.939 | Damaging | ≥0.4 is damaging; <0.4 tolerable |
| ClinPred | 0.999 | Damaging | ≥0.5 is damaging; <0.5 tolerable |
